# The Role of Connexin-43 in the Inflammatory Process: A New Potential Therapy to Influence Keratitis

**DOI:** 10.1155/2019/9312827

**Published:** 2019-01-21

**Authors:** Chu-Yang Xu, Wen-Song Zhang, Hong Zhang, Yan Cao, Hong-Yan Zhou

**Affiliations:** ^1^Department of Ophthalmology, China-Japan Union Hospital of Jilin University, Changchun 130033, Jilin Province, China; ^2^Department of Ophthalmology, The Second Hospital of Jilin University, Changchun 130000, Jilin Province, China

## Abstract

The studies outlined in this review highlight the relationship between inflammatory signaling molecules and connexin-43 (Cx43). Gap junction (GJ) channels and hemichannels (HCs) participate in the metabolic activity between intra- and extracellular space. Some ions and small molecules are exchanged from cell to cell or cell to extracellular space to affect the process of inflammation via GJ. We analyzed the effects of signaling molecules, such as innate immunity messengers, transcription factors, LPS, cytokine, inflammatory chemokines, and MMPs, on Cx43 expression during the inflammatory process. At the same time, we found that these signaling molecules play a critical role in the pathogenesis of keratitis. Thus, we assessed the function of Cx43 during inflammatory corneal disease. Corneal healing plays an essential role in the late stage of keratitis. We found that Cx43 is involved in wound healing. Studies have shown that the decrease of Cx43 can decrease the time of healing. We also report several Cx43 mimic peptides which can inhibit the activity of Cx43 Hc to mediate the releasing of adenosine triphosphate (ATP), which may in turn influence the inflammatory process.

## 1. Introduction

**Figure 1:**
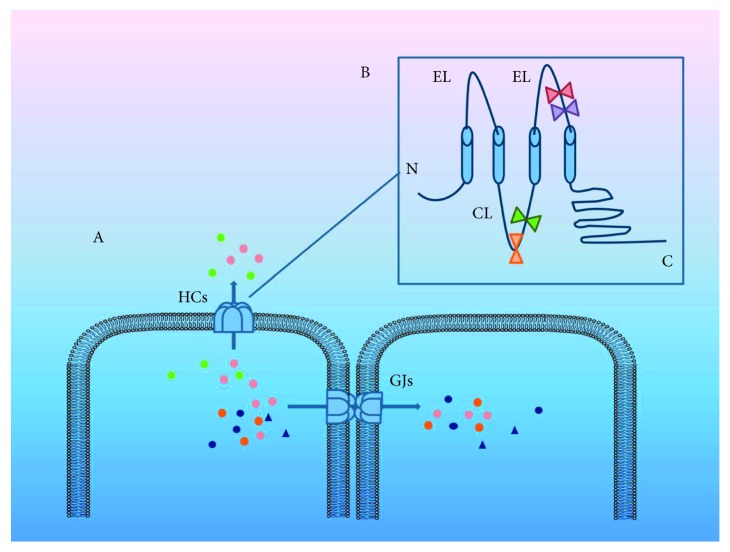
(A) Signal molecules, such as ATP, PGE2, glutamate, aspartate, and ions, transmit from cell to cell via GJs. Hemichannels (HCs) facilitate exchanges between intra- and extracellular compartments. Secondary messengers, small metabolites, and ions are involved in HC transmission. Thus, the diffusion of inflammatory signals can be carried out through GJs. (B) An HC is a tetraspan transmembrane (TM) protein with intracellular N- and C-terminals. HC has two extracellular loops and one cytoplasmic loop, which is the target of mimic peptide. In Cx43 HC, peptide 5 (red) and Gap27 (purple) target the extracellular loop. Meanwhile, L2 (green) and Gap19 (orange) target the cytoplasmic loop. These mimic peptides could regulate the activity of Cx43.

Gap junctions (GJs) appear at the cell plasma membrane and are formed by two interacting hemichannels (HCs) [1]. Each HC is composed of six protein subunits called connexins and pannexins, which are tetraspan transmembrane (TM) proteins with intracellular N- and C-terminals. HC has two extracellular loops (ELs) and one cytoplasmic loop (CL). There are more than 21 connexin (Cx) species in humans, and they are found in all tissues except differentiated skeletal muscle, erythrocytes, and mature sperm cells [2, 3]. HCs may consist of one or more different types of Cxs, while homotypic or heterotypic subunits of HCs may consist of various GJ channels space [4]. With the exception of intracellular communication, unopposed hemichannels (uHCs) can also express only on the cell surface, providing exchange between the intra- and extracellular compartment, such as autocrine and paracrine signaling molecules. Adenosine triphosphate (ATP), prostaglandin E2 (PGE2), glutamate, aspartate, and ions can be released from cells through the opening HCs [4–6]. Similarly, nutrient, fluorescent glucose derivative, or signaling molecule IP_3_ can also be transferred into cells via HCs [7] (Figure 1). GJs play an important role in the intercellular communication. This allows the intercellular transferring of the small molecules, under 1,000 daltons in size, such as secondary messengers, small metabolites, and ions [8]. HCs have been demonstrated to be regulated by diverse conditions including growth factors, proinflammatory cytokines, intracellular free Ca^2+^ levels, concentration of physiological extracellular cations, membrane potential, redox potential, protein phosphorylation, membrane stretch, alkalinization, acidification, hypoxia-reoxygenation, metabolic inhibition, and cellular nutrients (Figure 1) [7]. During the inflammatory process, GJs change with a high speed because of the short life of connexins [9].

It is concluded that both the connexin mRNA and protein are expressed in central corneal and limbal epithelia [10]. Connexins 26, 30.3, 31, 31.1, 33, 37, 43, and 50 are present in the central cornea, while Cxs 30, 40, 45, and 46 are found in the peripheral cornea [11]. In the normal cornea, Cx43 was mostly expressed in epithelium, from central cornea to the limbus, and anterior stroma. It is sure that Cx43 is important in regulating the growth and differentiation of the corneal cell; thus, Cx43 can affect corneal homeostasis [10, 12]. And the Cx43 antibody labels stromal keratocytes which are expressed in corneal fibroblasts [13]. Cx43 was found to participate in the development and normal physiology of the eye but is also equally involved in corneal inflammation [3].

## 2. Inflammation

**Figure 2:**
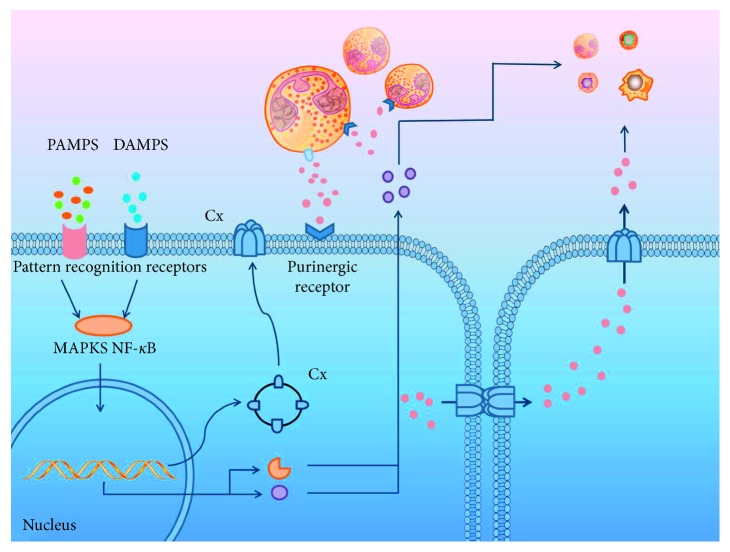
When inflammation occurs, many inflammatory signals can transport through GJs or HCs to produce a series of inflammatory reactions. For example, leukocytes infiltrate into the inflammatory tissue, releasing ATP via HCs, and then, ATP interacts with purinergic receptors on the cell surface to influence inflammation. Meanwhile, the purinergic receptors of leukocytes also recognize ATP. The following steps automatically appear: ATP affects adjacent cells through GJs and then it releases from cells through HCs, which leads more inflammation cells to the inflammatory tissue. In turn, inflammation may regulate the surface expression of GJs. PAMP and DAMP activate PRR regulating nuclear transcription processes through MAPKs, NF-*κ*B signaling pathways, which further affect transcription in the cell nucleus. This process can influence the expression of GJs and the releasing of chemokines and MMPs.

Inflammation is a complicated mechanism that protects an organism against pathogens and deleterious effects of cell damage. Inflammation involves infectious inflammation and sterile inflammation. The main step of the inflammation is the recruitment of neutrophils and macrophages, vasodilatation, increased permeability, and the production of inflammatory cytokines and chemokines [14, 15]. Connexin HCs play a role in mediating inflammation [3]. Studies have shown that in intestinal epithelial cells, connexin HCs were crucial to the invasion and dissemination of bacteria and virus [16]. Polymorphonuclear neutrophils (PMNs) are the first step in defending against infection. ATP as an autocrine or paracrine molecule releases from the cytoplasm into the extracellular space. ATP is involved in inflammatory diseases such as transplantation rejection, autoimmune disease, and bacterial infection [17, 18]. Purinergic receptors, which can be triggered by ATP, participate in the pathogenesis of various inflammatory diseases. This extracellular ATP can activate two classes of P2 receptors: P2X and P2Y purinergic receptors. ATP belongs to damage-associated molecular patterns (DAMPs), which is a signaling pathway that initiates macrophage activation. Through the Cx43 HC, ATP is released from the PMNs during immune activity [17–21], and ATP can then interact with P2X7 purinergic receptors at the cell plasma membrane to influence the inflammatory process. After releasing from neutrophils, ATP feedback via the P2X1 receptor signals neutrophils to accumulate in the inflammatory site. ATP can also transfer to the neighbor cell via gap junction to influence regulation of the cell [3] (Figure 2). Then, intracellular calcium is subsequently increased and stops neutrophil chemotaxis [17]. During infection, Toll-like receptors (TLRs), belonging to the family of pattern recognition receptors (PRRs), representing the first line of inflammation, play a key role in the host inflammation mechanism. Pathogen-associated molecular patterns (PAMPs) or DAMPs can interact with TLRs. Classical DAMPs are released by damaged cells, which can be found during injury and inflammation [22]. Typical PAMPs are peptidoglycans of most bacteria [3] and lipopolysaccharide (LPS) from Gram-negative bacteria [22]. Interestingly, the invading pathogen can result in the release of DAMPs [23]. After action by LPS, TLRs then subsequently facilitate signaling pathways, such as the transcription factor, nuclear factor (NF), *κ*-light-chain enhancer of activated B cells-*κ*B (NF-*κ*B), and mitogen-activated protein kinase (MAPK) pathways. Then, NF-*κ*B is transported to the nucleus, regulating the expression of cytokines, such as interleukin- (IL-) 1*β*, tumor necrosis factor (TNF)-*α*, and IL-6 [3, 22, 24, 25] (Figure 2). Neurotoxins, such as glutamate, nitric oxide, lactate, arachidonate, ammonia, reactive oxygen species, and calcium waves, may be transmitted from injured cells to healthy cells through GJs [26]. During the inflammatory process, some gene expression could be changed, such as up-regulation of Cx43 mRNA. Thus, the numbers of Cx43 HC are altered, which may lead to more ATP release and regulate cytokines releasing. ATP and cytokines which release from cells can attach more inflammatory cells to infiltrate into the injured tissue [27] (Figure 2).

### 2.1. The Regulation of Innate Immunity Process by GJs and Cx Channels

Once infected by a microorganism, innate immunity begins in the cornea. In this mechanism, TLRs play an important role in the innate immune cascade. TLRs cause a cascade process, which include neutrophil and antigen-presenting cell (APC) recruitment, bacteria phagocytosis, and some immune responses [28]. Activation of TLRs leads to innate immune responses, such as the secretion of inflammatory mediators, the killing of microbes, and the induction of adaptive immune responses [29]. Cx43 expression has been demonstrated in human DCs, and proinflammatory cytokines can influence the expression of Cx43 in the surface of DCs. Additionally, Cx43 participated in the release of cytokines and immunoglobulins [12]. Cx43 was found in macrophages in different tissues, which could partly increase the release of proinflammatory cytokines. Macrophages can communicate with other cells, such as DCs, endothelial cells, and T cells, via GJs. The functional expression of Cx43 occurs in the surface of neutrophils after stimulation by burn injury, wound healing, or spinal cord damage. These APCs could communicate with each other through GJs, which aids information sharing. This process contributes to amplify the immune response, which will lead to a cascade of immune cells [30]. Recruitment of neutrophils and T-cell activation can be controlled by Cx43 [3]. In the past, people believed that the cornea lacked APCs because the cornea is an immune-privileged site. However, recently, we have discovered that APCs, such as dendritic cells (DCs) and macrophages, are found both in mouse and human corneas after infection by the herpes simplex virus (HSV) [31]. In uninflamed cornea, the resident DCs are lacking the expression of major histocompatibility complex (MHC) class II and then obtain maturation after inflammation. Usually, these MHC class II-negative DCs reside in the epithelium and anterior stroma of the central cornea. After cornea is inflamed, MHC class II-positive DCs reside in the whole cornea [32]. Therefore, the upregulated expression of Cx43 protein in APCs may contribute to the inflammatory cells gathering into the inflamed corneas.

### 2.2. The Regulation of GJs and Cx Channels by Transcription Factors Process

MAPK pathways contain three core members: JNK, ERK, and p38 MAPK. To prevent bacterial infection, the transcription factor NF-*κ*B and the MAPKs JNK, ERK, and p38 contribute to the initiation of proinflammation, helping amplifying innate immunity. These signals can be rapidly propagated from infected to uninfected adjacent cells via GJs. This cell to cell propagation of proinflammatory signal communication through GJs was tested using 18*β*-glycyrrhetinic acid, a GJ inhibitor, to show IL-8 expression in bystander cells [33]. Activity of Cx43 gene promoter can be suppressed by activation of the JNK pathway. In an atrial fibrillation study, researchers found that downregulated Cx43 expression via activating the JNK pathway may be beneficial for treating atrial fibrillation in the elderly [34]. One study showed that paeoniflorin can alleviate ischemic brain edema, while Cx43 expression involved downregulation exerted through the JNK pathway activation [35]. The same regulation of Cx43 also occurs in the pancreatic tumor cells [36]. During *Pseudomonas aeruginosa* infection of airway epithelial cells, Cx43 was downregulated by JNK signaling, which may balance the inflammatory and apoptosis responses [16]. Interestingly, in bladder carcinogenesis, abnormally high expression Cx43 promoted the activation of the JNK pathway. The JNK and ERK pathways may aid Cx43 in the development of bladder cancer [37]. One study found that activation of the ERK signaling pathway resulted in increased Cx43 expression, which enhanced heterotopic ossification [38]. Connexin-43 expression was decreased in atrial myocytes after induction by macrophage migration inhibitory factor and ERK expression [39]. In H9c2 cells, after low-after-high glucose, MEK/ERK_1/2_ signal pathways activated, which resulted in a reduction of Cx43 expression [40]. In bone cells, the Cx43 HC was closed because of Cx43 phosphorylation, and it can be influenced by ERK pathway activation induced after the release of extracellular prostaglandin E2 [41]. The ERK pathway also induces Cx43 phosphorylation in acute cerebral ischemia [42]. In human endometrial stromal cells, IL-1*β* activates the p38 MAPK pathway to downregulate the Cx43 level [43].

### 2.3. The Regulation of GJs and Cx Channels by LPS

As a component of the wall of Gram-negative bacteria, LPS is a critical factor to induce inflammation. During LPS activation, GJ channels play an important role in transferring intracellular signals to spread infection and toxicity signals to neighboring cells and extracellular space [4]. In lung fibroblasts, LPS can induce apoptosis. Cx43 is increased after LPS stimulation. Through GJs, the apoptosis signals can be transmitted to adjacent cells one by one. The involvement of Cx43-composed GJs has been demonstrated in LPS-induced apoptosis [44]. The participation of Cx43-GJs in various apoptosis has been demonstrated in some studies. One study reported that Cx43 cardiomyocyte-mediated apoptosis is induced by silica nanoparticles [45]. Enteric glial Cx43 plays an important role in the enteric nervous system (ENS) pathophysiology. LPS upregulates Cx43 expression in enteric glia and secretes ATP, which are governed by connexin HCs [25]. During neuroinflammation, one study found that LPS induces Cx43 channel activity increase, while GJ communication has no change [46, 47]. Intracellular calcium increased due to the opening of Cx43 HCs [47]. Rat bone marrow mesenchymal stem cells showed that Cx43 decreased under hypoxia and serum deprivation conditions. However, with a low dose of LPS preconditioning, the decrease of Cx43 was attenuated, which may be due to the ERK signaling pathway [48]. Through the Rock1-MLC20 phosphorylation pathway, Cx43 takes part in the regulation of vascular permeability in sepsis, and the Cx43 mRNA and protein can be upregulated by LPS [49]. By contrast, during the LPS-induced inflammation, human cardiac fibroblasts were activated to alter Ca^2+^ signaling and downregulate Cx43. The intercellular communicated Ca^2+^ signaling of cardiac fibroblasts with dependents on the GJ channel [50]. In a rat model of X-linked Charcot-Marie-Tooth disease, Cx43-formed GJs presented in astrocytes appear to be remarkably reduced in LPS-injected mice [51]. In infectious keratitis, after the barrier function of the corneal epithelium is damaged, LPS was released into tear fluid and moves to stromal fibroblasts. LPS bind with soluble factors enhance the immune defense [52]. Toll-like receptor 4 is a main receptor for LPS in corneal stroma. In Gram-negative bacteria infectious cornea, NK cells secrete cytokines to stimulate TLR4 [53]. LPS increases TLR4, MMP-9, and cytokine expression in CFs. Overexpression of these products may cause corneal ulceration and perforation [54]. Thus, LPS was showed to regulate GJs in other tissues. And LPS can make a bad outcome in cornea. We hypothesis that regulating the expression of GJs may influence the inflammatory process caused by LPS.

### 2.4. The Regulation of Inflammatory Cytokines by GJs and Cx Channels

IL-1*β* as an early proinflammatory cytokine can cause inflammatory cascades. In CNS, astrocytes interconnect through GJ one by one, which can result in inflammatory pain. Some studies have shown that after injection with proinflammatory cytokines, IL-1*β* appears to be an important factor causing the low expression of Cx43 [55]. In a disease of the CNS, multiple sclerosis (MS), a progressive series of inflammation can reduce Cx43 GJs in astrocytes. First, type 1 T helper (Th1) cells secrete interferon- (IFN-) *γ* into the intercellular space, and then, microglia activated by IFN-*γ* release humoral factors that decrease the Cx43 protein level in astrocytes. Among these humoral factors, IL-1*β* is a major factor that suppresses the function of GJs in astrocytes [56]. Endometrial receptivity can be influenced by inflammation, and Cx43 is known to be important for embryonic implantation. IL-1*β* is secreted from endometrial tissue macrophages and stromal cells. Then, IL-1*β* decreases Cx43 expression, which can be mediated via the ERK-MAPK signaling pathway [43]. In vascular smooth muscle cells, IL-1*β* inhibits the expression level of Cx43 [57]. TNF-*α*, another important inflammatory cytokine, participates in the regulation of Cx43 levels. During inflammation in vascular smooth muscle cells, TNF-*α* directly inhibits Cx43 via the JNK pathway, which leads to apoptosis of vascular smooth muscle cells [57]. The Cx43 formed GJ in DCs, while TNF-*α* combined with IL-1*β* could advance the GJ communication [30]. The current study demonstrated that a mixture of TNF-*α* and IFN-*γ* activated the JNK pathway, leading to a significant decrease of Cx43 and its GJ function in spinal astrocytes [58]. In liver epithelial cells, TNF-*α* induces a downregulation of both Cx43-formed GJIC and Cx43 protein and mRNA levels [59]. In astrocyte cultures of mice, TNF-*α* evoked a large upregulation of Cx43 HC activity but not GJIC. After injecting a mixture of TNF-*α* and IL-1*β*, the GJC was reduced, but the Cx43 HC was active [60]. Similarly, in mice spinal astrocytes, injection with a mixture of TNF-*α* and IFN-*γ* led to a significant downregulation of Cx43 GJIC. Its influence can be influenced by blocking the JNK pathway [61]. Interestingly, in the model of guinea pig hearts, high extracellular calcium with TNF-*α* can modulate conduction velocity by increasing Cx43 [62]. In fungal and bacterial keratitis, proinflammatory cytokines are found in tears, suggesting cytokines are participated in the process of infectious keratitis [63]. IL-1*β* is produced by mucosal epithelial cells of the ocular surface and immune cells, and it appears to the ocular surface to mediate corneal injury after infecting with fungal and Gram-negative bacteria. Likewise, IL-6 and IL-8 present in tears during microbes invade the cornea. Gram-negative keratitis cause increasing circulation of NK cells. NK cells help IFN-*γ* response on interaction with dendritic cells [53]. It is demonstrated that, in fungal keratitis, mature DCs release chemokines to encourage the secretion of TNF-*α* IL-4, IL-5, and IL-13 [64]. In the rat corneal deep stromal wound model, with the treatment of Gap27, granulocytes and macrophages were accumulated and late gene expression of TNF-*α* and TGF*β*1 increased [65]. TNF-*α* induces granulocyte-macrophage colony-stimulating factors in corneal stromal, which promote immune cells infiltration. During corneal infection, in response to TNF-*α*, the Cx43 protein was reduced, which may influence the corneal homeostasis [66]. Though the relationship between cytokines and GJs needs more research, we still think regulating GJs and Cx channels is good for inflammation in infectious cornea.

### 2.5. The Regulation of Chemokines by GJs and Cx Channels

During the inflammation process, the activation of chemokines could induce PMN in lesions. In the condition of corneal transplantation, chemokines are released to induce adaptive responses [67]. We found that Cx43 was involved in the secretion of chemokines during inflammation. Stromal cell-derived factor 1 (SDF1) is also known as C-X-C motif chemokine 12 (CXCL12). In astrocytes, Cx43 activated the Ca^2+^-cAMP-PKA signaling pathway to upregulate the release of CXCL12, which leads to neuropathic pain [68]. Another study found that injected TNF-*α* in the spinal cord can induce the substantial release of chemokines CCL2 and CXCL1 through upregulating the Cx43 level of astrocytes, which contributes at maintaining late-phase neuropathic pain and enhancing synaptic transmission in mice [60]. Cx43 is important for regulating B cell motility and migration. The stromal cell-derived factor 1 could help B cell precursors stay in bone marrow, and Cx43 is also critical for chemokine-mediated Rap 1 activation and CXCL12-directed migration [69]. The same is true in human bone marrow stromal cells, Cx43 controlled CXCL12 secretion, and transcription. The Ca^2+^ transmits through GJs to regulate CXCL12 secretion. Intercellular communication of the bone marrow microenvironment can be negatively impacted by LPS, which leads to CXCL12 downregulation [70]. In turn, CXCL12 could also regulate Cx43. In breast cancer cells, CXCL12 leads to the phosphorylation of Cx43 through activation of protein kinase C. Thus, low levels of CXCL12 can downregulate Cx43 expression and its phosphorylation [71]. A study of corneal grafts found that CXCL1 is the early chemokine, while CXCL9 and CXCL10 are late chemokines [67]. Several studies have reported that CCL2, CCL3, and CXCL2 are crucial chemokines to the corneal disease. In herpetic stromal keratitis, CXCL10 is secreted after infection by HSV-1. Releasing of CXCL10 contributes to reduction in inflammation in herpetic stromal keratitis. In the absence of CXCL10, secretion of CXCL9 is a compensatory way to alleviate corneal disease [72]. In the *P. aeruginosa* infected cornea, the CXC chemokine receptor-2 (CXCR2) is important for neutrophils' recruitment. The major ligands of this receptor are CXCL1, CXCL2 in mouse, and CXCL8 in human [73]. In other tissues, GJs and HCs could regulate the expression of chemokines. Thus, we suppose this regulation also exists in infectious cornea.

### 2.6. The Regulation of MMPs by GJs and Cx Channels

Matrix metalloproteases (MMPs) are a family of zinc-dependent proteases that regulate cellular migration, proliferation, adhesion, apoptosis [74], and growth factor activity [75] as well as participating in immunity, inflammatory progress, and wound healing [76]. MMPs take part in a series of inflammatory cascades. In the first line of inflammation, MMPs help the recruitment of leukocytes and degrade the mediate extracellular matrix (ECM) to promote leukocyte infiltration [77] (Figure 2). Then, MMPs regulate inflammatory mediators, such as cytokines and chemokines. Lastly, because of MMPs, cell death is induced by interrupting the communication between cells and surrounding ECM [78]. MMPs participate in acute inflammation together with Cx43 channels [77].

Some studies have demonstrated that MMP activity correlates with the expression of connexin. During the condition of hypoxia, the expression of H9c2 cardiomyocyte Cx43 becomes progressively weaker. However, when we use MMP inhibitors and block ERK_1/2_ signaling, the reduction of Cx43 can be slower [79]. During heart failure, ECM is remodeled by MMPs, which are regulated by the tissue inhibitor of matrix metalloproteinases (TIMPs). The increasing MMP-2 showed that induce angiogenesis to avoid heart failure. And moderate TIMP-2 translates pro-MMP-2 to MMP-2, while excess TIMP-2 leads inhibition of MMP-2. In the TIMP-2-/- aortic banding heart, the MMP-9 and MMP-14 were increased, resulting in extensive downregulation of Cx43 [80]. In a canine ventricular fibrillation (VF) model, the ratios of MMP-2/TIMP-2 were higher, and the Cx43 level was significantly decreased [81]. One study demonstrated that MMP-2 and MMP-9 increased after myocardial infarction could destroy the connection between myocardial cells leading to the reduction of Cx43 and gap junction remodeling [82]. However, the consequence was different in diabetic kidneys. In one study, H_2_S was found to be a modulator of diabetic renovascular remodeling and kidney dysfunction. It can mitigate the expression and activity of MMP-9. It can also decrease Cx43 through the MMP-9-mediated pathway [83]. In cornea, MMPs participate in degradation and remodeling ECM and involve in corneal injury and repair. Several reports reveal that MMP-3, MMP-1, and MMP-9 are released at sites of corneal wounds [84]. In inflammatory cornea, MMP-14 promotes corneal hemangiogenesis and lymphangiogenesis [85]. During keratitis, MMP-8, MMP-9, and MMP-13 are known to initiate extracellular matrix degradation and allow effector cells following injured tissue [86]. There are few articles that report the regulation of MMPs and Cx43 in keratitis. However, as other tissues, we suppose this regulation also exists in cornea. It provokes more research.

## 3. The Role of Cx in the Progression of Keratitis

GJs participate in corneal diseases, such as chemical injuries, infection, and hypersensitive immune responses. After cornea injury, the corneal healing occurs partly via GJ mediation. GJs play an important role in corneal homeostasis because they can regulate cornea cell growth and differentiation [12]. Cx43 is the most common GJ protein expressed in the corneal epithelium and in the stroma [87, 88]. Some studies have shown that Cx43 was upregulated both in the epithelium and stroma during human corneal disease [12]. During inflammation, Cx43 is expressed in the activated leukocytes. Furthermore, Cx43 is involved in the releasing of cytokines [89]. Thus, the Cx43 protein may contribute to infectious keratitis.

Infectious keratitis is caused by microbial pathogens such as *Pseudomonas aeruginosa*, *Staphylococcus aureus*, or *gonococcus*. With the damage of the corneal epithelium, the first line of protection against infection, microbial pathogens, can invade the stroma, causing microbial infections of the cornea, which may lead to visual impairment or blindness [90]. Both the pathogen and its components, such as LPS, released from Gram-negative bacteria can penetrate and develop infection in the stroma. Then, resident keratocytes transform into stromal fibroblasts (CFs) which can increase the inflammatory response [52]. Thus, corneal stromal fibroblasts play a critical role in the sequence of inflammation and corneal healing.

TLRs belonging to pattern recognition receptors (PRRs) initiate the stimulation of the early host defense against pathogens [22, 91]. TLR-2 and TLR-4 are usually expressed in the CFs, and they have the ability to recognize the LPS. At sites of epithelial abrasion, LPS flowing from tear fluid stimulates TLRs. In corneal fibroblasts, LPS induces the activation of MAPK by phosphorylating it [92]. Bacteria, fungi, and viruses mediate cell signaling through NF-*κ*B and MAPK pathways. Cytokines and chemokines transcribe after the activation of the NF-*κ*B and MAPK pathways [93–95], and CFs produce cytokines and chemokines, including IL-6, monocyte chemotactic protein- (MCP-) 1, and IL-8 [52, 54, 96]. However, IL-1*β*, the essential chemokine in the bacterial clearance and recruitment of neutrophils, is secreted by neutrophils themselves [52, 97]. TNF-*α* is a proinflammatory cytokine which induces corneal inflammation. It can affect GJIC of human corneal fibroblasts by reducing the expression of Cx43 through the JNK signaling pathway without the effect on the amount of Cx43 mRNA. Eventually, TNF-*α* will cause a series of reactions in the corneal fibroblasts, inducing adhesion molecules such as IL-8 and MMPs [13]. In addition to TNF-*α*, IL-1*β* can also induce corneal fibroblast to produce MMPs, which cause the degradation of stromal collagen fibrils [98]. After the appearance of MMPs in the corneal stroma, corneal ulceration occurs [99]. The corneal ulceration results from the stromal melting, which is caused by the interaction between corneal fibroblasts and infiltrated leukocytes [100, 101]. Corneal fibroblasts synthesize and secrete MMP-1, MMP-2, MMP-3, MMP-9, and MMP-14 after corneal injury and keratectomy [98, 102].

In the late stage of keratitis, the wound healing system largely helps the injured cornea reestablish homeostasis. The corneal epithelium has four to six nonkeratinized stratified squamous epithelial cells [9, 103]. At the migrating edges of open wounds, the expression of Cx43 was reduced, which is good for uncoupling of adjacent connexons between cells, leading to wound gap closing [65]. The wound healing process requires numerous growth factors and cytokines, such as transforming growth factor, platelet-derived growth factor, fibroblast growth factor, epidermal growth factor, and insulin-like growth factor [27, 104]. In a rabbit model, Cx43 was increased after laser photorefractive keratectomy [105]. Further, after chemically burned and infected corneas, Cx43 was found to be upregulated [12]. Furthermore, studies have demonstrated that upregulating the expression of Cx43 contributes to wound healing in injured corneas [103, 106], and corneal wound healing shares some common processes with skin healing [103], including inflammation, myofibroblast differentiation, ECM deposition, and eventually development of fibrosis. However, the greatest difference is that the cornea is an avascular organ [107], although corneal repaired growth factors largely overlap with those of the skin [108–111]. We suppose that corneal GJs may play the same role in wound healing as those in the skin. Cx43 reduction is associated with the upregulation of TGF-*β* and collagen *α*1 and downregulation of the inflammatory mediators chemokine (C-C motif) ligand 2 (CCL2) and TNF-*α* [112–114]. Cx43 phosphorylation and TGF-*β*1 aid fibroblast transformation into myofibroblasts, resulting in wound recovery [9, 115]. At epidermal wound margin, the expression of Cx43 decreased while the Cx43 expression in dermal fibroblasts was upregulated. Expression of functional proteins is firmly associated with cell migration. Increased expression of Cx43 blocks cell migration during wound healing [116, 117]. One study demonstrated that Cx43 deficiency can improve reepithelialization, increase proliferation, activate dermal fibroblasts, and promote the expression of ECM mediators. However, it has no impact on collagen deposition [117]. In other studies, deficiency of Cx43 was shown to enhance ECM through increasing collagen type I, collagen type III, and MMP-2 [114]. The expression of Cx43 is significantly different in chronic wounds. Chronic wounds, such as diabetic foot ulcers, pressure ulcers, and venous leg ulcers, are common around the world. In chronic diabetic foot ulcer, the Cx43 expression level increased largely at the wound edge [118, 119]. Some reports suggest that the Cx43 level increased because of the high glucose level, leading to the repression of filopodial extensions and fibroblast migration rates [120]. For pressure ulcers, a model of ischemia-reperfusion injury in fibroblasts showed increasing expression and activation of Cx43 and cell death [121]. In many corneal wounding models, reduction of Cx43 translation protein significantly reduces edema and inflammation and speeds the rate of epithelial recovery [122]. Taken together, upregulation of the expression of Cx43 can delay wound repair. Thus, reducing the Cx43 level is beneficial to the process of wound healing.

## 4. Connexin Inhibitors Negatively Impact the Process of Inflammation through Mediating Cx43 Expression

In recent years, there has been increasing interest in the use of GJ inhibitors to mediate various clinical conditions. Connexin mimetic peptides inhibit GJIC via the corresponding extracellular loop of connexins, and they also can inhibit the ATP releasing from cell and Ca^2+^ entry into the cell from extracellular space [123]. Gap19 is a mimetic peptide that interacts with the Cx43 CT and selective inhibitor toward Cx43 HC [124, 125]. Divalent ions are known to trigger the opening of connexin HCs. Therefore, mediating extracellular ATP can influence connexin HC activity. TAT-Gap24 and TAT-Gap19 can block extracellular release of ATP [126] (Figure 1). In cultured human gingival fibroblasts, Gap27 and TAT-Gap19 block Cx43 through the ERK_1/2_ pathway, which depends on reduced ATP signaling [127]. Peptide 5 is a cysteine-containing mimetic peptide of Cx43. It attenuated HC activity at a low concentration while blocking GJ at a higher concentration [128, 129] (Figure 1). In an in vitro model, P5 peptide suppressed endotoxin-induced release of ATP and high-mobility group box 1, inflammatory mediators, by innate immune cells. In vivo, the P5 peptide protective effect issues from ischemia/reperfusion injury and microbial infections [130]. Some studies have suggested that innate immune cells and nonimmune cells may communicate through Cx43GJ channels to mediate inflammation [130, 131]. Thus, the P5 peptide may offer protection by disrupting this communication between immune and nonimmune cells [130]. The action of P5 peptide contributes to the treatment of retinal injury, chronic diseases, and other central nervous system diseases [132]. L2 peptide inhibits Cx43 Hc by affecting a sequence in the CL of Cx43 (Figure 1). This peptide closes Cx43GJ channels during low pH/ischemic conditions [133]. The interaction between the CT and CL results in Cx43 Hc closing. Thus, the function of L2 induces a residual state in a low level of conductance condition [124, 134].

Both Gap26 and Gap27 are highly selective mimetic peptides toward Cx43 Hc [128]. Gap26 and Gap27 block unopposed Cx43 Hc through mimicking on the first and second extracellular loops of Cx43 [60, 135, 136] (Figure 1). Gap26/27 inhibits Cx43 Hc combined chemical/electrical activation [128]. Gap27 can block function of Cx43, which leads to less fibroblast cell death [137]. Gap27 can prevent cell death as well as preventing the influence of healthy cells by dead cells [9, 138, 139]. Injected with Gap27, mouse models showed increasing migration of keratinocytes and fibroblasts, leading to wound healing. However, this effect is not suitable for diabetic wound healing [9, 140]. One study discovered the relationship between the expression of spinal Cx43 and cancer pain. When treated with Gap26 to inhibit the activity of Cx43 Hc, the mouse pain tolerance improved [68]. In corneal inflammation, after treatment with scGap27 and Gap27, the release of TNF-*α* was decreased; meanwhile, the release of IL-6 was reduced after scGap27 treatment. Gap27 can accelerate the corneal epithelial wound healing, which may be used to promote corneal epithelial healing when persistent corneal ulcers occur, limbal stem cells are deficient, and dry eye syndrome appears [65]. The alpha-carboxy terminus 1 (*α*CT1) peptide is a 25-amino acid peptide from the C-terminus of connexin-43 (Cx43) [141]. The tight junction zonula occluded 1 (ZO-1) binds with Cx43 C-terminus end [142]. During wound healing, interaction between the C-terminus of Cx43 and ZO-1 would influence cellular communication and GJ remodeling. However, *α*CT1 could block this binding, helping cornea wound healing and reducing scar tissue formation. *α*CT1 can affect early migration of corneal healing and epithelial-mesenchymal transformation pathway genes [103]. In a streptozocin (STZ) model of type I diabetes, data suggested suppression of the inflammatory response after treatment with *α*CT1. In research, TNF-*α* decreased with *α*CT1 treatment, and it corresponded to the corneal wound closure rate. The inflammatory reduction can lead to epithelial migration and proliferation, which contribute to corneal wound healing [143]. In addition, *α*CT1 also applies in skin and brain [144, 145].

In addition to connexin mimetic peptide, we found some GJ inhibitors as well. 18*α*-glycyrrhetinic acid (GA) and 18*β*-GA were found to inhibit different GJs without being connexin subtype-specific. Heptanol and octanol, long-chain alcohols, inhibited GJs in the crayfish giant axon, rat glial cells, insect cells, cardiac cells, stomach, and pancreatic epithelial cells. Flufenamic acid inhibits Cx43GJ, but it is not selected, and RNA interference can be applied to inhibit connexin expression. Cx43 expression has also been suppressed via RNA interference [134]. Several studies have used RNA interference to mediate Cx43, such as bone marrow stromal cells [146], bronchial fibroblasts [147], rat renal tubular epithelial cells [148], ovarian cancer cell lines [149], and pulmonary endothelial cells [150]. Reducing Cx43 mRNA has been demonstrated to downregulate inflammation and helps to improve healing [134].

Thus, we set out a hypothesis of the potential Cx43 therapy in inflammatory keratitis. As expression of Cx43 is observed in corneal epithelium and stroma, it has been suggested that Cx43 might be involved in infectious cornea. With the damage of the epithelial barrier, then followed by the inflammatory process in the corneal stroma, keratitis occurs. As are the cases with other inflammatory tissues, Cx43 and proinflammatory cytokines are involved in keratitis. Both the GJ and HC of Cx43 contribute to the inflammatory process in many tissues and, as cytokines and Cx43 are increased in infectious cornea, regulation of Cx43 might contribute to the development of this disease. Evidence supporting this concept comes from a study that demonstrated that Gap27 has the potential to promote inflammatory cell migration and accumulation in deep corneal stromal [65]. Importantly, some studies report that Cx43 is influenced by the LPS-induced expression of inflammatory cytokines in corneal stroma. These studies, taken together with those described in the previous paragraph about the relationship between Cx43 and inflammation, suggest that Cx43 and GJIC might contribute to and therefore be potential therapeutic targets for infectious cornea.

## 5. Conclusions

Keratitis may cause serious damage to the cornea. It leads to opacity, ulceration, perforation of the cornea, and finally may deprive eyesight. The main treatment of infectious keratitis is antibacterial and antiviral, at present. Current studies have shown that GJs are involved in inflammatory responses and play an important role in this process. Bacteria and viruses are common pathogens affecting the corneal microenvironment and causing inflammation. There are similarities between the progression between keratitis and inflammation in other organs. We analyzed the change of Cx43 expression during various inflammatory responses. Unfortunately, until now, few studies have been performed examining the relationship between keratitis and Cx43 expression. According to the relationship in other inflammatory processes, we assume that the keratitis reaction may also affect the expression of Cx43. Mediating Cx43 is also helpful for the repair of keratitis. Therefore, future challenges will be to better understand the relationship between corneal inflammation and Cx43 expression. Here, we explain the possible use of Cx43 inhibitors to treat keratitis. Related studies may evaluate whether Cx43 inhibitors may be applied in the treatment of keratitis. Thus, future studies may determine whether Cx43 Hc inhibitors contribute to keratitis inflammatory control and corneal wound healing as well as whether inhibiting other types of GJs could help different kinds of corneal inflammation.
